# Effect of severe renal impairment on the pharmacokinetics of brigatinib

**DOI:** 10.1007/s10637-021-01095-5

**Published:** 2021-03-20

**Authors:** Neeraj Gupta, Michael J. Hanley, David Kerstein, Meera Tugnait, Narayana Narasimhan, Thomas C. Marbury, Karthik Venkatakrishnan

**Affiliations:** 1 Millennium Pharmaceuticals, Inc., a wholly owned subsidiary of Takeda Pharmaceutical Company Limited, 40 Landsdowne Street, Cambridge, MA 02139 USA; 2 Present Address: Anchiano Therapeutics, Cambridge, MA USA; 3 Present Address: Spectrum Pharmaceuticals, Inc., Cambridge, MA USA; 4 Present Address: Verastem Oncology, Needham, MA USA; 5 Orlando Clinical Research Center, Orlando, FL USA; 6 Present Address: EMD Serono, Inc., Billerica, MA USA

**Keywords:** Brigatinib, Anaplastic lymphoma kinase, Pharmacokinetics, Renal impairment

## Abstract

*Background* Brigatinib, a next-generation anaplastic lymphoma kinase (ALK) inhibitor, targets activated, mutant forms of ALK and overcomes mechanisms of resistance to the ALK inhibitors crizotinib, ceritinib, and alectinib. Brigatinib is approved in multiple countries for treatment of patients with ALK-positive non–small cell lung cancer. Based on population pharmacokinetic (PK) analyses, no dosage adjustment is required for patients with mild or moderate renal impairment. *Methods* An open-label, single-dose study was conducted to evaluate the PK of brigatinib (90 mg) in patients with severe renal impairment (estimated glomerular filtration rate < 30 mL/min/1.73 m^2^; n = 8) and matched healthy volunteers with normal renal function (estimated glomerular filtration rate ≥ 90 mL/min/1.73 m^2^; n = 8). Plasma and urine were collected for the determination of plasma protein binding and estimation of plasma and urine PK parameters. *Results* Plasma protein binding of brigatinib was similar between patients with severe renal impairment (92 % bound) and matched healthy volunteers with normal renal function (91 % bound). Unbound brigatinib exposure (area under the plasma concentration-time curve from time zero to infinity) was approximately 92 % higher in patients with severe renal impairment compared with healthy volunteers with normal renal function. The renal clearance of brigatinib in patients with severe renal impairment was approximately 20 % of that observed in volunteers with normal renal function. *Conclusions* These findings support a brigatinib dosage reduction of approximately 50 % in patients with severe renal impairment.

Trial registry: Not applicable.

**Supplementary Information:**

The online version contains supplementary material available at 10.1007/s10637-021-01095-5.

## Introduction

Brigatinib is a next-generation anaplastic lymphoma kinase (ALK) inhibitor that targets a broad range of ALK mutations, including L1196M, F1174C/V, I1171N, and G1202R [1, 2]. Mechanisms of resistance to the ALK inhibitors crizotinib, ceritinib, and alectinib were overcome by brigatinib in nonclinical studies, and brigatinib was found to be a more potent and selective inhibitor of native EML4-ALK compared with these three agents [3]. In the pivotal phase 2 ALTA trial of patients with locally advanced or metastatic ALK-positive non–small cell lung cancer (NSCLC) that progressed on crizotinib, brigatinib 180 mg qd with a seven-day lead-in at 90 mg was associated with an independent review committee-assessed confirmed objective response rate (ORR) of 56 % and median progression-free survival (PFS) of 16.7 months with a median follow-up of 24 months [4]. For patients with baseline brain metastases, brigatinib demonstrated a confirmed intracranial ORR of 67 % and median intracranial PFS of 18.4 months. Based on these data, brigatinib was approved for the treatment of patients with metastatic ALK-positive NSCLC with progressive disease on or with intolerance to crizotinib [5]. In a subsequent phase 3 trial (ALTA-1L) in patients with ALK + NSCLC who had not previously received an ALK inhibitor, PFS was significantly longer among patients who received brigatinib than among those who received crizotinib (24-month PFS: blinded independent review committee-assessed, 48 % vs. 26 %; hazard ratio, 0.49 [95 % confidence interval [CI], 0.35–0.68]; *P* < 0.0001; investigator-assessed, 56 % vs. 24 %; 0.43 [0.31–0.61]; *P* < 0.0001) [6], leading to expanded approval for first-line use.

The recommended dosing regimen for brigatinib consists of a seven-day lead-in of 90 mg orally once daily (qd) followed by 180 mg qd [5]. This regimen provides a favorable benefit-risk profile by providing a lower starting dose to reduce the frequency of rare early-onset pulmonary events, followed by a higher dose to provide the potential for greater efficacy [7, 8]. Brigatinib is rapidly absorbed, with a median time to reach maximum plasma concentration (T_max_) of 1 to 3 h postdose [7]. Steady-state systemic exposure is dose proportional over the dose range of 60 mg to 240 mg qd [7], and the mean plasma elimination half-life is 25 h [5]. Brigatinib has modest drug accumulation with repeat dosing, with a geometric mean accumulation ratio after repeat dosing of 1.9 to 2.4 [5, 7]. Brigatinib is primarily metabolized by cytochrome P450 (CYP) 3A4, with minor contribution by 2C8 in vitro. In the human absorption, distribution, metabolism, and excretion study, 65 % and 25 % of the administered dose was recovered in feces and urine, respectively. Unchanged brigatinib accounted for 41 % and 86 % of the total radioactivity in feces and urine, respectively. Therefore, both hepatic and renal pathways of elimination contribute to the overall clearance of brigatinib in humans [5].

Brigatinib is intended for chronic use in patients with ALK + NSCLC, some of whom may have impaired renal function of varying degrees, especially if they have been previously treated with agents known to be associated with renal toxicity. During development, patients with mild or moderate renal impairment, defined by an estimated glomerular filtration rate (eGFR) of 30 to < 90 mL/min/1.73 m^2^, were allowed to enroll into clinical studies. Based on population pharmacokinetic (PK) analyses, mild or moderate renal impairment were not found to impact brigatinib exposure, thereby indicating that no dose adjustment is required for these patients [9]. Because patients with severe renal impairment (eGFR < 30 mL/min/1.73 m^2^) were excluded from the pivotal studies, the present study evaluated the PK of brigatinib in patients with severe renal impairment and matched healthy volunteers with normal renal function (≥ 90 mL/min/1.73 m^2^) to inform dosing recommendations in this patient population.

## Methods

### Study design

This was an open-label, parallel-group study that enrolled patients with severe renal impairment and matched healthy volunteers with normal renal function. The study consisted of a 28-day screening period and a single nine-day inpatient treatment period (day − 1 to day 8). On the morning of day 1, each participant received a single oral dose of brigatinib 90 mg under fasting conditions.Participants were required to abstain from food for approximately 10 h prior to study drug administration and for approximately four hours postdose, with the exception of the standardized snack. Due to the high prevalence of diabetes in patients with severe renal impairment, the standardized snack (not high fat or high calorie) was given to all participants at 2.5 h postdose. Water was permitted ad libitum except for one hour prior to dosing and for approximately one hour postdose. Participants underwent end-of-study procedures and were discharged from the clinical site on day 8.

Approval for this study was obtained from the institutional review board of the study site (IntegReview, Austin, Texas, USA) and all participants provided written informed consent. This study was conducted in accordance with current Good Clinical Practice, the Declaration of Helsinki, the International Council for Harmonisation guidelines, and all applicable regulatory requirements.

### Participants

Healthy volunteers with normal renal function (eGFR ≥ 90 mL/min/1.73 m^2^) were matched to patients with severe renal impairment by age (± 10 years), sex, body mass index (BMI; ±15 % at screening), and if possible, smoking habits. Patients with severe renal impairment were defined as those with an eGFR < 30 mL/min/1.73 m^2^. eGFR was calculated using the Modification of Diet in Renal Disease equation (MDRD) [10]. Estimated creatinine clearance was based on the Cockcroft-Gault equation.

Eligible participants included men or women of nonchildbearing potential aged 18 to 80 years with a BMI of 18.0 to 45.0 kg/m^2^ and a minimum weight of 50.0 kg at screening. Other key inclusion criteria required a nonsmoker or a smoker who was willing to smoke five or fewer cigarettes per day during the inpatient stay. Healthy volunteers with normal renal function were free from any clinically significant abnormality based on medical history, vital signs, physical examination, 12-lead electrocardiogram, and laboratory evaluation at screening.

Patients with severe renal impairment may have had related medical conditions consistent with their disease, such as hypertension and diabetes, that were stable for at least three months, but they were excluded if they had a functioning renal transplant or fluctuating or rapidly deteriorating renal function. Use of over-the-counter drugs or herbal supplements, with the exception of occasional acetaminophen (≤ 2000 mg/day) and vitamins (≤ 100 % recommended daily allowance), was not permitted within 72 h of study drug administration nor was any investigational or prescription drug within 30 days except chronic stable medications taken by patients with renal impairment. Participants were required to abstain from grapefruit and grapefruit-containing products, pomegranate, pomelo, star fruit, poppy seeds, Seville oranges, quinine-containing drinks or foods, and caffeine-containing beverages for 72 h before study drug administration.

### Assessments

#### Plasma protein binding

Blood samples were collected at 2, 8, and 24 h after brigatinib administration to determine the plasma protein binding of brigatinib.

#### Pharmacokinetic measurements

Blood samples were collected before brigatinib administration (predose) and at 0.5, 1, 1.5, 2, 2.5, 3, 4, 6, 8, 12, 24, 36, 48, 60, 72, 96, 120, 144, and 168 h after brigatinib administration to determine plasma brigatinib concentrations. Urine was collected predose and during intervals (0–8, 8–12, 12–24, 24–48, 48–72, 72–96, 96–120, and 120–168 h) after brigatinib administration to determine urine brigatinib concentrations. Urine samples were subsequently diluted 50:50 (v:v) urine:isopropanol at the clinical site.

### Bioanalytical methods

#### Plasma protein binding assay

Free brigatinib in plasma samples was separated from protein-bound brigatinib by dialyzing the samples across a semipermeable dialysis membrane (8000-Da mass cutoff) using Thermo Scientific™ Single-Use RED (rapid equilibrium dialysis) Plates. For each RED dialysis unit, an aliquot (200 µL) of plasma was transferred into the donor chamber and an aliquot (350 µL) of warm (37 °C) phosphate buffered saline (PBS) was transferred into the receiver chamber of the dialysis unit. The RED devices were incubated at 37 °C with gentle shaking (250 rpm on a rotator shaker) for six hours. A 125-µL aliquot of plasma and 150-µL aliquot of PBS were removed from the dialysis unit and matrix-matched to a final composition of 50:50 (v:v) plasma:PBS, in Rain-X® treated 96-well plates. The matrix-matched samples were immediately frozen at − 80 °C. The protein binding assay and sample analysis were conducted at Charles River Laboratories, Inc. (Worcester, MA, USA). Samples were analyzed for brigatinib concentrations using liquid chromatography with tandem mass spectrometry (LC-MS/MS) methods that have been previously reported [11] using a dual-range assay with a lower limit of quantitation of 0.100 ng/mL and an upper limit of quantitation of 500 ng/mL.

#### Pharmacokinetics assay

Plasma and urine samples were analyzed by Charles River Laboratories, Inc. (Worcester, MA, USA) by LC-MS/MS, as previously reported [11]. Plasma samples were analyzed using a dual-range assay with a lower limit of quantitation of 0.100 ng/mL and an upper limit of 2500 ng/mL [11]. Urine samples were analyzed using a single-range assay with a lower limit of quantitation of 25.0 ng/mL and upper limit of 2500 ng/mL [11].

### Safety

Adverse events were recorded throughout the study. Physical examinations were performed at screening and on days − 1 and 8; height, weight, and BMI were collected only at screening. Vital signs were recorded at screening; on day − 1; at 0.5, 1, 2, 3, 4, 6, 8, 12, 24, 36, 48, 60, 72, 96, 120, and 144 h after brigatinib administration; and on day 8. Electrocardiograms were conducted at screening, day − 1, before the brigatinib dose, 3 and 48 h after brigatinib, and on day 8. Clinical laboratory evaluations were performed at screening and on days − 1 and 8.

### Pharmacokinetic data analysis

Individual participant plasma concentration-time profiles were analyzed via noncompartmental analysis methods using Phoenix WinNonlin (version 6.4) to estimate PK parameters. Plasma PK parameters calculated for brigatinib included maximum observed plasma concentration (C_max_); T_max_; area under the plasma concentration-time curve from time zero to the time of the last measurable concentration (AUC_0 − last_); AUC from time zero to infinity (AUC_0−∞_); terminal elimination half-life (t_1/2_); apparent oral clearance (CL/F); and apparent volume of distribution (V/F). The fraction of unbound brigatinib in plasma was determined using the following formula: % free (unbound) = [free brigatinib concentration measured in buffer after RED]/[total concentration measured in plasma] X 100. Unbound brigatinib plasma PK parameters (except unbound CL/F and V/F, which were calculated as total CL/F or total V/F divided by the overall mean fraction unbound) were calculated using the following formula: unbound PK parameter = (parameter based on total concentrations) x (individual participant’s overall mean fraction unbound value). Urinary PK parameters included the amount of brigatinib excreted from time zero to time t (Ae_0 − t,_), fraction of the dose excreted (%) from time zero to time t (Fe_0 − t_), and renal clearance (CL_R_).

### Statistical analysis

Descriptive statistics, including number of observations (n), arithmetic mean, standard deviation (SD), coefficient of variation (CV%), median, minimum, and maximum, were calculated for each time point for brigatinib plasma concentrations in each study group. Arithmetic mean; SD; CV%; median, minimum, and maximum values; and geometric mean and geometric CV% were calculated for all PK parameters except T_max_, which was reported as median (minimum, maximum). Statistical comparisons of brigatinib PK parameters (unbound) were made using a one-factor analysis of variance, and 90 % CIs for the geometric mean ratios (GMRs) were calculated for the comparison of C_max_, AUC_0 − last_, and AUC_0−∞_ between study groups. Descriptive statistics were used to present safety outcomes, including number and percentage of participants with adverse events.

## Results

### Demographics and baseline characteristics

A total of 16 participants were screened and enrolled in the study, including eight patients with severe renal impairment and eight matched healthy volunteers with normal renal function. All participants completed the study, and no participants were excluded from any analyses. Demographic and baseline characteristics were generally similar in both study groups (Table 1). Age ranged from 47 to 70 years in both groups, with a mean of 60 years in healthy volunteers with normal renal function and 62 years in patients with severe renal impairment. Most participants were male, white, and had never smoked. Baseline eGFR ranged from 90 to 122 mL/min/1.73 m^2^ for healthy volunteers with normal renal function and from 10 to 26 mL/min/1.73 m^2^ for patients with severe renal impairment. Results were similar when renal impairment was categorized by eGFR using the MDRD equation or creatinine clearance using the Cockcroft-Gault equation.

**Table 1:** Demographics and baseline characteristics

Characteristic	Normal Renal Function (n = 8)	Severe Renal Impairment (n = 8)
Age, mean (SD), years	59.8 (9.1)	61.8 (7.3)
Male, n (%)	6 (75.0)	6 (75.0)
Race, n (%)		
White	5 (62.5)	7 (87.5)
Black or African American	3 (37.5)	1 (12.5)
Ethnicity, n (%)		
Hispanic or Latino	1 (12.5)	3 (37.5)
Not Hispanic or Latino	7 (87.5)	5 (62.5)
BMI, mean (SD), kg/m^2^	32.5 (3.4)	33.1 (3.9)
Smoking habit, n (%)		
Never smoked	7 (87.5)	7 (87.5)
Current smoker	1 (12.5)	0
Former smoker	0	1 (12.5)
Serum creatinine, mean (SD), mg/dL	0.8 (0.1)	3.5 (1.1)
eGFR, mean (SD), mL/min/1.73 m^2^	107.4 (10.2)	18.3 (4.4)
eGFR range	90–122	10–26

BMI, body mass index; eGFR, estimated glomerular filtration rate; SD, standard deviation

### Plasma protein binding of brigatinib

**Fig. 1:**
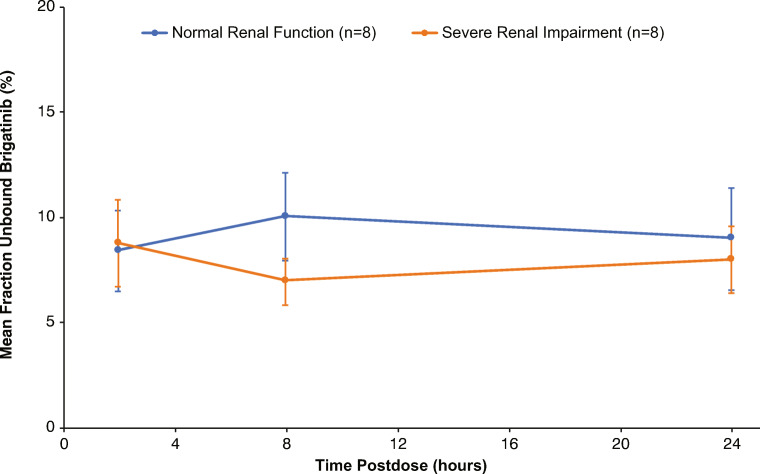
Mean (standard deviation) fraction unbound for brigatinib at 2, 8, and 24 h postdose by renal function group

Blood samples were collected at 2, 8, and 24 h postdose to assess the plasma protein binding of brigatinib. The mean fraction unbound for brigatinib was similar across the three time points for both renal function groups, indicating that protein binding was not concentration-dependent (Fig. 1). Accordingly, the plasma protein binding data were averaged across the three time points for each participant to calculate a mean unbound value for the subsequent derivation of unbound plasma PK parameters for brigatinib. There was no effect of severe renal impairment on brigatinib plasma protein binding, as the overall mean (SD) fraction bound was 91 % (2.2 %) for healthy volunteers with normal renal function and 92 % (1.4 %) for patients with severe renal impairment.

### Plasma pharmacokinetic parameters for total brigatinib

Evaluation of PK parameters for total brigatinib showed higher C_max_ concentrations and AUC values for patients with severe renal impairment versus healthy volunteers with normal renal function. Geometric mean (CV%) for total brigatinib C_max_ was 471.1 (27.2 %) ng/mL for patients with severe renal impairment and 362.4 (32.1 %) ng/mL for healthy volunteers with normal renal function (GMR [90 % CI]: 1.30 [1.00–1.68]). Brigatinib total AUC_0−∞_ and AUC_0 − last_ were more than 100 % higher (AUC_0−∞_ GMR [90 % CI]: 2.19 [1.75–2.74]; AUC_0 − last_ GMR [90 % CI]: 2.02 [1.65–2.48]) in patients with severe renal impairment compared with healthy volunteers with normal renal function.

Median (range) T_max_ was 2.5 (1.0–3.0) and 2.0 (1.0–4.0) hours in the severe renal impairment and normal renal function groups, respectively. The mean t_1/2_ of brigatinib after a single dose was approximately 54 h in patients with severe renal impairment and 42 h in healthy volunteers with normal renal function.

### Plasma pharmacokinetics of unbound brigatinib

**Fig. 2:**
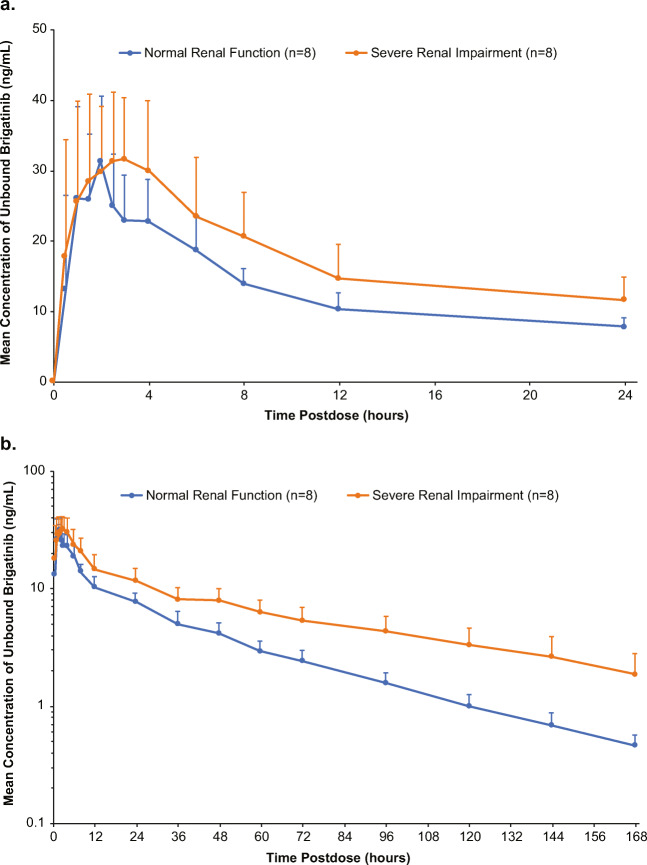
Mean (standard deviation) unbound brigatinib plasma concentration-time profiles from (**a**) 0 to 24 h postdose, linear scale and (**b**) 0 to 168 h postdose, log-linear scale

Plasma PK parameters for unbound brigatinib showed similar trends to those for total brigatinib. Unbound plasma drug concentration-time curves are provided in Fig. 2a (0–24 h postdose) and b (0–168 h postdose). After a single oral dose of brigatinib 90 mg, patients with severe renal impairment had higher unbound plasma brigatinib concentrations compared with healthy volunteers with normal renal function. PK parameters for unbound brigatinib are shown in Table 2. Geometric means for unbound brigatinib C_max_ were generally comparable across the two groups (GMR [90 % CI]: 1.14 [0.90–1.44]), whereas unbound brigatinib AUC values were higher in patients with severe renal impairment compared with healthy volunteers with normal renal function (Table 2). Specifically, unbound brigatinib AUC_0 − last_ and AUC_0−∞_ were 77 % (GMR [90 % CI]: 1.77 [1.43–2.20]) and 92 % (GMR [90 % CI]: 1.92 [1.52–2.42]) higher, respectively, in patients with severe renal impairment versus healthy volunteers with normal renal function (Table 3).

**Table 2:** Plasma pharmacokinetic parameters for unbound brigatinib

Parameter^a^	Normal Renal Function (n = 8)	Severe Renal Impairment (n = 8)
C_max,u_ (ng/mL)	32.29 (25.1)	36.76 (29.0)
Range	23.9–45.3	25.2–55.6
AUC_0 − last,u_ (h·ng/mL)	613.1 (22.4)	1086 (27.3)
Range	438–847	726–1544
AUC_0−∞,u_ (h·ng/mL)	641.4 (22.2)	1230 (30.6)
Range	460–883	799–2017
CL/F,_u_ (L/h)	140.3 (22.2)	73.15 (30.6)
Range	102–196	44.6–113
V/F,_u_ (L)	8327 (30.4)	5532 (31.4)
Range	5898–13,744	3556–8914

AUC_0−∞,u_, unbound area under the plasma concentration-time curve from time zero to infinity; AUC_0 − last,u_, unbound area under the plasma concentration-time curve from time zero to the time of the last measurable concentration; CL/F,_u_, unbound apparent oral clearance; C_max,u_, unbound maximum observed plasma concentration; V/F,_u_, unbound apparent volume of distribution

^a^ Data presented as geometric mean (geometric % coefficient of variation)

**Table 3:** Comparison of unbound brigatinib plasma pharmacokinetic parameters

Parameter	Comparison	LS Mean (SE)	Geometric LS Mean Ratio (90 % CI)
C_max,u_ (ng/mL)	Severe renal impairment vs. Normal renal function	0.130 (0.1330)	1.14 (0.90–1.44)
AUC_0 − last,u_ (h·ng/mL)		0.572 (0.1229)	1.77 (1.43–2.20)
AUC_0−∞,u_ (h·ng/mL)		0.652 (0.1311)	1.92 (1.52–2.42)

AUC_0−∞,u_, unbound area under the plasma concentration-time curve from time zero to infinity; AUC_0 − last,u_, unbound area under the plasma concentration-time curve from time zero to the time of the last measurable concentration; CI, confidence interval; C_max,u_, unbound maximum observed plasma concentration; LS, least squares; SE, standard error

### Urinary pharmacokinetics of brigatinib

The mean (SD) fraction of the administered brigatinib 90 mg dose excreted from 0 to 168 h postdose was 8.4 % (4.2) in patients with severe renal impairment and 18.8 % (4.0) in healthy volunteers with normal renal function, thereby representing a reduction of approximately 50 % (Online Resource 1). In addition, brigatinib renal clearance in patients with severe renal impairment was approximately 20 % of that observed in healthy volunteers with normal renal function.

### Safety

Two patients with severe renal impairment reported three treatment-emergent adverse events. One patient had both a serious adverse event hip fracture and a mild skin abrasion; both were considered unlikely to be related to study drug by the investigator. The second patient experienced an influenza-like illness, which was assessed as mild and possibly related to study drug by the investigator. Both patients completed the study. One additional patient with severe renal impairment had a clinically significant elevated systolic blood pressure postdose on day 1, but also had a medical history of hypertension. No healthy volunteers with normal renal function reported adverse events. There were no treatment-emergent adverse events related to clinical laboratory results reported, and no participant had clinically significant laboratory values or electrocardiogram findings during the study.

## Discussion

The clearance of the ALK inhibitor brigatinib is characterized by a major contribution of cytochrome P450-mediated metabolism and a minor contribution of renal clearance. Accordingly, the purpose of this study was to evaluate the impact of severe renal impairment on brigatinib PK to complement population PK model-based assessments of brigatinib PK in mild or moderate renal impairment. Although unbound maximum plasma concentrations of brigatinib were comparable between renal function groups following single-dose oral administration, total systemic exposure of unbound brigatinib (AUC_0−∞_) was approximately 92 % higher in patients with severe renal impairment compared with healthy volunteers with normal renal function. Severe renal impairment had no effect on brigatinib plasma protein binding. The renal clearance of unbound brigatinib was approximately five-fold lower in patients with severe renal impairment compared with that in healthy volunteers with normal renal function. The observation of an approximately 92 % higher total systemic exposure of unbound brigatinib in severe renal impairment supports a brigatinib dosage reduction of approximately 50 % (i.e., from 180 mg to 90 mg or from 90 mg to 60 mg) for patients with severe renal impairment to result in unbound systemic exposures comparable to those achieved in patients with normal renal function.

The data from this study add to the information on the PK and dosing of brigatinib across clinical contexts of use. Brigatinib displays dose-linear and time-independent PK, with biphasic elimination and a mean plasma half-life of approximately 25 h [5, 7]. Approximately 25 % of an oral dose of brigatinib is excreted unchanged in the urine [12]. Mild or moderate renal impairment was not associated with clinically meaningful effects on brigatinib PK based on the results of a population PK analysis; consequently, no dosage adjustments are required in patients with eGFR ≥ 30 mL/min/1.73 m^2^ [9]. In addition, a high-fat meal was shown to have no effect on the total exposure (AUC) of brigatinib, and thus the drug can be administered without regard to meals [13]. Brigatinib is primarily metabolized by CYP3A4, with minor contribution by 2C8 in vitro [11]. Systemic exposures (AUC) of brigatinib were found to increase by 101 % with coadministration of strong CYP3A inhibitors and decrease by 80 % with strong CYP3A inducers; therefore, concomitant use of these agents with brigatinib is not recommended [11]. When concomitant use of brigatinib with a strong CYP3A inhibitor cannot be avoided, the brigatinib dose should be reduced by approximately 50 % [11]. In contrast, coadministration of brigatinib with a strong inhibitor of CYP2C8 did not meaningfully affect brigatinib systemic exposures (AUC), and no dose modifications are therefore needed when brigatinib is coadministered with strong CYP2C8 inhibitors [11].

The results of this study indicate that both renal and nonrenal clearance of brigatinib are impacted in severe renal impairment. Renal clearance of brigatinib in patients with severe renal impairment was 20 % of that in healthy volunteers with normal renal function. As C_max_ was not meaningfully different between the renal function groups, it appears that bioavailability is not affected by renal impairment. Considering that renal clearance accounts for approximately 25 % of overall clearance of brigatinib, it can thus be derived that nonrenal clearance of brigatinib in severe renal impairment is approximately 50 % lower than in the setting of normal renal function. Although the precise mechanism of the effects of renal impairment on the nonrenal clearance of brigatinib is unknown, the results of this study provide the scientific basis for appropriate dosage of brigatinib in order to achieve a favorable benefit versus risk profile during treatment of patients with ALK + NSCLC who have severe renal insufficiency.

In conclusion, this study showed that brigatinib exposure is higher in patients with severe renal impairment compared with healthy volunteers with normal renal function and supports an approximate 50 % dose reduction in this patient population, as reflected in the prescribing information [5, 14].

## Supplementary Information

ESM 1 (PDF 107 kb)

## Data Availability

The data sets, including the redacted study protocol, redacted statistical analysis plan, and individual participant data supporting the results reported in this article, will be made available within three months from initial request, to researchers who provide a methodologically sound proposal. The data will be provided after de-identification, in compliance with applicable privacy laws, data protection, and requirements for consent and anonymization.
