# Supplementary material for: Effect of severe renal impairment on the pharmacokinetics of brigatinib
Source: Invest New Drugs. 2021 Mar 20;39(5):1306–14. doi: 10.1007/s10637-021-01095-5 (PMC8426299; doi:10.1007/s10637-021-01095-5)
Supplement: Supplementary file 1 — (PDF 107 kb) [file 10637_2021_1095_MOESM1_ESM.pdf]

**Online Resource 1. Urine pharmacokinetic parameters for brigatinib**

| Parameter[a] | Normal Renal Function (n=8) | Severe Renal Impairment (n=8)[b] |
|---|---|---|
| $Ae_{0–168}$ (mg) | 16.90 (3.57) | 7.58 (3.77) |
| Range | 11.58–21.77 | 3.92–14.36 |
| $Fe_{0–168}$ (%) | 18.78 (3.96) | 8.42 (4.19) |
| Range | 12.9–24.2 | 4.36–16.0 |
| $CL_R$ (mL/min) | 41.45 (11.55) | 9.17 (3.09) |
| Range | 27.5–63.7 | 5.40–13.4 |

$Ae_{0–168}$, amount of brigatinib excreted from time zero to 168 hours, $CL_R$, renal clearance, $Fe_{0–168}$, fraction of the dose (%) excreted from time zero to 168 hours.

[a] Data presented as mean (standard deviation)

[b] n=7 for $Ae_{0–168}$ and $Fe_{0–168}$, as one participant did not have urine collected after 72 hours postdose
